# Measuring the impact of health problems among adults with limited mobility in Thailand: further validation of the Perceived Impact of Problem Profile

**DOI:** 10.1186/1477-7525-6-6

**Published:** 2008-01-21

**Authors:** RoseAnne Misajon, Julie F Pallant, Lenore Manderson, Siriporn Chirawatkul

**Affiliations:** 1 School of Political and Social Inquiry, Monash University, 900 Dandenong Rd, Caulfield East, Victoria 3145, Australia; 2 School of Rural Health, University of Melbourne, 49 Graham Street, Shepparton, 3630, Victoria, Australia; 3 School of Psychology, Psychiatry & Psychological Medicine, Monash University, 900 Dandenong Rd, Caulfield East, Victoria 3145, Australia; 4 Faculty of Nursing, Khon Kaen University, Khon Kaen, Thailand

## Abstract

**Background:**

The Perceived Impact of Problem Profile (PIPP) was developed to provide a tool for measuring the impact of a health condition from the individual's perspective, using the ICF model as a framework. One of the aims of the ICF is to enable the comparison of data across countries, however, relatively little is known about the subjective experience of disability in middle and low-income countries. The aim of this study was to assess the validity of the Perceived Impact of Problem Profile (PIPP) for use among adults with a disability in Thailand using Rasch analysis.

**Methods:**

A total of 210 adults with mobility impairment from the urban, rural and remote areas of northeast Thailand completed the PIPP, which contains 23 items assessing both impact and distress across five key domains (Self-care, Mobility, Participation, Relationships, and Psychological Well-being). Rasch analysis, using RUMM2020, was conducted to assess the internal validity and psychometric properties of the PIPP Impact subscales. Validation of the PIPP Impact scales was conducted by comparing scores across the different response levels of the EQ5D items.

**Results:**

Rasch analysis indicated that participants did not clearly differentiate between 'impact' and 'distress,' the two aspects assessed by the PIPP. Further analyses were therefore limited to the PIPP Impact subscales. These showed adequate psychometric properties, demonstrating fit to the Rasch model and good person separation reliability. Preliminary validity testing using the EQ5D items provided support for the PIPP Impact subscales.

**Conclusion:**

The results provide further support for the psychometric properties of the PIPP Impact scales and indicate that it is a suitable tool for use among adults with a locomotor disability in Thailand. Further research is needed to validate the PIPP across different cultural contexts and health conditions and to assess the usefulness of separate Impact and Distress subscales.

## Background

The International Classification of Functioning, Disability and Health (ICF) was developed by the World Health Organization (WHO) [1] to provide a standard, unified language and framework to describe health and health-related states. A specific aim of the ICF is to enable the comparison of data across countries and health care disciplines. To achieve this, two areas need to be further addressed.

The first is that until recently, much of the research worldwide has focused on etiology, treatment and epidemiology. An advancement of the ICF, compared with previous classification tools, was to incorporate contextual factors, including physical and social environmental factors as well as personal factors (e.g. age, education, coping styles), into a model of functioning and disability. Consequently the ICF adopted a biopsychosocial approach, integrating conventional medical and social models. However, limited attention has been given to non-clinical, particularly social and personal aspects of health, disability and illness [2].

In addition to the need to further elaborate on these contextual factors [1], there is a need for a clear statement regarding where the ICF is placed in relation to the extensive literature on subjective well-being and quality of life. In its current form, the ICF provides an extensive framework for the objective dimensions of human life, and articulates in detail physical aspects of health and functioning. Some have found it a useful framework to compare the content of health-related quality of life measures [3]. Greater clarity is required however as to how the ICF might integrate both objective and subjective dimensions to provide a more complete and comprehensive classification of functioning, disability and health [4]. Indeed, the current shift in health and disability research places increasing emphasis on the social construction of disability, and on the individual's subjective experience of his or her health condition.

The second gap in the literature is that research on non-communicable and chronic disease has been conducted primarily in Western Europe and North America, and relatively little is known about the subjective experiences of disability in middle and low-income countries. Concern has been raised about the application of Western notions of well-being, illness and disability across different cultures, including the adaptation of health and health-related quality of life measures [5-8]. Cultural context plays an important role in the experience of disablement, and disability cannot be considered in isolation from factors such as ethnicity, gender, and religion. Little research has been conducted to explore the interrelationship of these factors. The RESILIENCE project addressed these two gaps.

The RESILIENCE project (**RE**search into **S**ocial **I**nclusion, **L**ocomotor **I**mpairment and **E**mpowerment through **N**etworking, **C**ollaboration and **E**ducation) was a large interdisciplinary, multi-country project which considered the contextual factors which impact upon the subjective experience of physical impairments in Australia and Southeast Asia. Both qualitative and quantitative research methods were used to explore the personal and social environmental factors that contributed to disability and disablement in the different country and social settings (see [9,10]). One of the countries in which the project was conducted was Thailand [11]).

Thailand has a national population of 63 million (31 million male, 32 million female) [12], two thirds of whom live in rural areas. As in many countries worldwide, the elderly population is increasing (expected 7 million in 2010), due to higher life expectancy (69.1 years) (most recent data available, Thailand [13]). A 1991 survey indicated 1.1 million people with disabilities, equivalent to 1.8 percent of the then total population of 57 million. The majority had physical disabilities, and resided in the poor northeast region, followed by the north of Thailand [14]. A second survey conducted in 1999 produced similar findings [15]. Studies have been undertaken in Thailand examining health problems of people with disabilities, particularly stroke, amputation or paraplegia [16-24]; however little work has been conducted on the experience of living with disability (but see [25]).

As part of the RESILIENCE project, we developed the Perceived Impact of Problem Profile (PIPP [9]) as a relatively short, self-report instrument to assess, from the individual's point of view, the impact and distress associated with a health condition, rather than the person's ability to perform a particular task [9]. It has been recommended that the ICF be considered during the development phase of instruments, as this assists in achieving a stronger basis for international comparability [26]. For the development of the PIPP, selection of the domains was guided in part by the ICF, but also by a review of existing measures and a series of qualitative interviews. One of the concerns of existing measures is that most have been developed in English-speaking countries, leaving researchers with the options of either developing a new measure or translating an existing measure [8], with consequent difficulties associated with salience and comparability. The PIPP addresses this concern in that the 23 items were developed on the basis of baseline ethnographic data, conducted in collaboration with researchers from Australia, Malaysia and Thailand. In all three countries, wording and content were chosen carefully to ensure that the activities described were suitable across different cultural contexts, for both men and women, and across different age groups. The instrument was designed to be generic to allow for comparisons across health conditions.

We have previously published our analysis of the PIPP, validating its use among adults with mobility impairment in Australia [9]. Overall, the five subscales (Self-care, Mobility, Participation, Relationships, and Psychological Well-being) showed adequate psychometric properties, with both impact and distress subscales demonstrating good fit to the Rasch model. In this paper, we use Rasch analysis to assess the validity of the Perceived Impact of Problem Profile (PIPP) for use among adults with a locomotor disability in Thailand.

## Methods

The study was conducted in urban, rural and remote areas of Khon Kaen Province in the Northeast Region (Isaan), the setting of the Thai arm of the RESILIENCE study. The Isaan region, with a population of 19 million, is the largest of four regions in Thailand. The majority of the population are of Lao descent and ethnicity, and those living in rural areas are among the poorest in Thailand. Ethics clearance was granted by Khon Kaen University and The University of Melbourne.

### Participant recruitment and data collection

A modified cluster sampling method was employed, following stratification into urban, sub-urban, rural and remote areas. In total, 38 villages were randomly identified. In each village, the headman was asked to list names of persons "having difficulty in movement" (in Isaan, *pai sai ma sai yak*). A researcher then visited the persons identified, and during the visit, used a snowball technique to identify other persons having difficulty in movement. All persons who were invited to participate agreed to be involved in the study. The participant criteria included age (≥ 18 years), ability to communicate well enough to give informed consent, and willingness to participate as well as experience of a condition causing difficulty in movement. These difficulties were ones defined by the participants, reflecting lay understandings of impairment and ability, and did not use pre-selected clinical criteria. The recruitment area was extended to contiguous villages if a village was not large enough to identify seven suitable participants. The study was conducted between September 2003 and August 2004.

The socio-demographic and health profile of participants is listed in Table 1. In summary, 210 respondents in 40 villages, of whom 70 percent were female and 30 percent male, participated in the quantitative survey. The average age was 64 years old, with the majority living in rural areas (73 percent) and having completed primary school level education only (96 percent). Approximately half of the participants were married, while a third were separated, divorced or widowed. Two-thirds had one or more child(ren) living in the house, and the majority had three or more adults living in the one house (77 percent). Almost all were Buddhist (99 percent), and of either Thai or Isaan ethnicity (60 and 40 percent respectively). The most common cause of mobility problems was sickness/illness (34 percent), and the average duration of locomotor impairment was 10 years.

**Table 1:** Socio-demographic and health profile of participants (N = 210)

**Variable**	**%**
**Age**	
- Range [24–89 yrs]	
- Mean yrs & SD [64.1 ± 14.7]	
**Gender**	
- Male	29.5
- Female	70.5
**Residency**	
- Rural	72.9
- Urban	27.1
**Marital status**	
- Never married	11.0
- De facto	3.3
- Married	51.9
- Separated/divorced./widowed	33.8
**Child/ren in household**	
- Range [0–5 children]	
- Mean & SD [1.2 ± 1.1]	
- no children	30.0
- 1 child	31.0
- 2 or more children	39.1
**Adults in household**	
- Range [1–9 adults]	
- Mean & SD [3.5 ± 1.5]	
- 1 adult only	8.1
- 2 adults	15.2
- 3 or more other adults	76.7
**Religion**	
- Buddhist	99.0
- Christian	1.0
**Ethnicity**	
- Thai	59.5
- Isaan	40.6
**Education level**	
- Range [0–10 yrs]	
- Mean yrs & SD [5.1 ± 4.2]	
- Primary (1–6 yrs)	96.2
- Secondary (7–12 yrs)	3.8
- Tertiary (≥ 13 yrs)	0.0
**Health Profile**	
**Cause of mobility problems**	
- Sickness/Illness	34.3
- Accident	16.7
- Since birth	2.9
- Don't know	15.2
- Others	31.0
**Other health problems**	
- Hypertension	10.5
- Diabetes	19.5
- Arthritis	3.8
- Stroke	1.0
- Heart disease	1.4
- Lung disease (inc.asthma, TB)	4.3
- Others	19.5
**Duration of mobility problems**	
- Range [1–68 yrs]	
- Mean years & SD [10.3 ± 11.3]	

### Materials

#### Perceived impact and associated distress related to mobility impairment

The Perceived Impact of Problem Profile (PIPP) was developed as a relatively short, self-report instrument to assess both the impact and the distress of health problems from the individual's perspective [9]. The development of the 23 items in the PIPP was guided in part by the WHO's ICF [1]. The domains include self-care, mobility, relationships, participation, and psychological well-being. For each item, respondents were asked to rate on a 6-point scale (a) 'how much impact has your current health problems had on [item of function or activity]'; and (b) 'How much distress has been caused by the impact of your health problem on [same item of function or activity]'. The 6-point scale was anchored on either end by 'no impact' and 'extreme impact' for the Impact scale and by 'no distress' and 'extreme distress' for the Distress scale. High scores indicate greater impact. In the current Thai study, the PIPP was interviewer administered, although the instrument can be administered by an interviewer or self completed. Instrumentation was developed in English. It was then translated into Thai, drawing on ethnographic data collected during early phases of the study, with the intent and precise meanings of terms discussed and pre-tested during training. Clarity was confirmed through back-translation prior to pilot testing and finalizing the instrument. The initial Thai language version of the PIPP was pilot tested in the study area with a series of interviews conducted with adults with mobility limitations.

#### Other measures

Participants were also asked to complete information regarding their socio-demographic background (age, gender, years of formal education, ethnicity, religion, marital status, and household size), health background (cause and duration of mobility problems, co-morbidities), and current health status as measured by the EQ-5D [27]. The EQ-5D, developed by the EuroQoL group, is a standardized, validated generic instrument and is available in Thai, Malay, Bahasa Indonesia, and Chinese [28], and was included in the study not only to provide a health status profile of participants, but also for the purposes of validating the PIPP instrument. On the EQ-5D, respondents are asked to describe their own health according to five domains: self-care, mobility, usual activities, pain/discomfort, and anxiety/depression.

The EQ-5D and PIPP were pretested with 20 people with a disability in periurban communities of Khon Kaen City to establish clarity of the questions and the sequence of the items.

### Statistical analysis

To assess the psychometric properties of each PIPP Impact subscale, the relevant items for each were subjected to Rasch analysis using the RUMM2020 software [29]. Rasch analysis, which was originally developed by Georg Rasch [30] is increasingly being used in the health and psychological sciences to guide the development and validation of the measurement tools [31]. It provides a detailed analysis of many aspects of a scale, including the response format, fit of items and persons, item bias, internal consistency, dimensionality and targeting.

The procedures adopted in this study are consistent with those conducted in the preliminary validation of the PIPP in an Australian sample (for details see [9]; for a more detailed description of Rasch analysis procedures, see [32]). The response format was evaluated by inspection of the thresholds. Disordered thresholds would indicate that respondents had difficulty consistently discriminating among response options. Categories were collapsed if required to achieve satisfactory model fit. The overall fit to the model was assessed using the item-trait chi-square interaction statistic, with a Bonferroni adjustment to the probability value. Non-significant chi-square values indicated model fit. Individual person-fit and item-fit were also assessed using chi square statistics and fit residual values. Residual values between ± 2.5 were considered to indicate adequate fit to the model. The Person Separation Index (PSI) is equivalent to Cronbach alpha and provides an estimate of the internal consistency reliability, with values above .8 considered adequate. Item bias can occur when different groups within the sample display different response patterns to a particular item, despite being equivalent in terms of the underlying characteristic being measured. To identify any possible item bias across gender and age, differential item functioning (DIF) was assessed.

Preliminary analysis indicated high levels of concordance in responses to the PIPP Impact items (associated with function and experience) and PIPP Distress items (associated with feelings), despite linguistic differentiation. People tended to give the same value to each item for both impact and distress. For the purposes of this paper, therefore, we chose to evaluate only one set of subscales, those relating to impact of health problems. Rasch calibrated PIPP Impact subscales scores were exported to SPSS Version 12 for further statistical analysis to assess the construct validity of the subscales. Non-parametric techniques were used due to the non-normal distribution of scores for a number of the scales. Spearman correlation coefficients were generated to assess the intercorrelations among the PIPP Impact subscales. Mann-Whitney U tests and Kruskal-Wallis Tests were used to compare PIPP subscale scores across the various levels of responses to the EQ5D items.

## Results

### Rasch analysis of PIPP Impact Subscales

Preliminary inspection of the threshold map (not shown) for the PIPP Impact items indicated disordered thresholds for many of the items. This suggests that respondents experienced difficulty in utilizing the full 6-point response scale, but instead typically used only four response points. All items were therefore rescored by collapsing categories, with a change in scoring from 012345 to 011223.

The four Self-care items showed adequate fit to the model after Bonferroni adjustment to the alpha level (overall item-trait interaction chi square = 19.65, df = 8, p = .01) with good person separation reliability (PSI = .89). No item showed misfit (see Table 2) and no DIF was detected for either gender or age.

**Table 2:** Individual item fit statistics for PIPP Impact scale items

		**Location**	**SE**	**Fit Residual**	**DF**	**Chi Sq**	**DF**	**Prob**
**Self-care**								
**4**	*Wash self*	0.13	0.11	-1.21	105.45	7.96	2	0.02
**5**	*Use toilet*	-1.24	0.12	0.27	106.18	1.07	2	0.59
**6**	*Dress self*	-0.06	0.11	-0.12	106.18	6.99	2	0.03
**7**	*Feed self*	1.18	0.13	1.21	106.18	3.33	2	0.19
**Mobility**								
**9**	*Sit/stand*	0.26	0.13	1.80	125.48	1.72	2	0.42
**11**	*Use vehicle*	-0.21	0.13	1.26	98.39	0.38	2	0.83
**12**	*Move – house*	0.38	0.11	-1.64	125.48	3.85	2	0.15
**13**	*Move – neighbourhood*	-0.43	0.12	-0.67	117.64	5.38	2	0.07
**Relationship**								
**14**	*People in authority*	-0.86	0.12	0.48	86.58	0.52	2	0.77
**15**	*Neighbours & friends*	-0.21	0.11	-0.10	97.94	3.28	2	0.19
**16**	*Relatives*	-0.10	0.11	-1.01	98.65	8.27	2	0.02
**17**	*Close relationship*	1.18	0.13	1.15	90.84	2.82	2	0.24
**Participation**								
**8**	*Assist family members*	0.40	0.1	1.06	117.44	0.07	2	0.96
**18**	*Family activities*	0.15	0.10	0.32	121.9	5.31	2	0.07
**19**	*Community activities*	-0.20	0.10	0.71	119.67	0.43	2	0.81
**20**	*Activities you enjoy*	0.32	0.10	1.09	110.01	2.19	2	0.33
**21**	*Work*	-0.67	0.11	-0.39	86.97	1.38	2	0.50
**Psychological**								
**1**	*Overall life satisfaction*	-0.30	0.10	1.26	144.62	1.44	2	0.49
**2**	*Moods & feelings*	-0.15	0.10	0.26	140.73	0.62	2	0.73
**3**	*Confidence*	0.05	0.10	1.36	131.4	0.03	2	0.98
**22**	*Live independently*	0.23	0.09	-0.95	145.4	6.13	2	0.05
**23**	*Reliance on others*	0.170	0.09	0.07	143.84	2.04	2	0.36

SE = Standard Error, DF = degrees of freedom, ChiSq = Chi square, Prob = probability All probability values non-significant after Bonferroni adjustment for the number of items in each subscale.

Good fit to the model was achieved for the five Mobility items (overall item-trait interaction chi square = 17.0, df = 10, p = .07); however significant DIF for age was detected for items *carry *and *move around the house*. Removal of the item *carry *resulted in no DIF for any item, improved model fit (overall item-trait interaction chi square = 11.33, df = 8, p = .18) and good person separation (PSI = .85).

The four Relationship items showed good model fit (chi square = 14.89, df = 8, p = .06) and adequate person separation reliability (PSI = .88). No items showed misfit (Table 2) and there was no significant DIF for age and gender.

A non-significant overall item-trait interaction chi square was obtained for the five Participation items (chi square = 9.39, df = 10, p = .50), suggesting good model fit. No items showed misfit (see Table 2), and there was no significant DIF for age. However, significant DIF by gender was found for *participation in family activities*, with males showing a greater likelihood of endorsing this item than females. Removal of the item resulted in fit to the model, however the PSI value dropped from .79 to .72, indicating a undesirable reduction in the person separation reliability of the scale. Given that the DIF noted for the item was relatively minor, and that the original overall model fit was very good, it was decided to retain the item in the scale for further investigation.

The five Psychological items revealed adequate person separation reliability (PSI = .83) and good fit to the model (overall item-trait interaction chi square = 10.27, df = 10, p = .42). No items showed misfit (see Table 2) and there was no DIF for gender or age.

### Correlations among PIPP subscales

Table 3 shows the Spearman correlation coefficients (rho) among the Rasch calibrated scores for the PIPP subscales. The strongest correlation was between the impact on Mobility and Self-care (rho = .69), with the lowest occurring between Relationships and Psychological well-being (rho = .39). The pattern of quite strong correlations among the subscales is supportive of the construct validity of the PIPP, given the expected relationship among the various aspects assessed. None of the correlations were so high as to indicate redundancy, with the highest of .69, indicating only 48% shared variance.

**Table 3:** Spearman correlation coefficients among PIPP Impact subscales

Impact subscales	Self-care	Mobility	Relation	Particip
Self-care				
Mobility	.693			
Relationships	.449	.422		
Participation	.561	.672	.524	
Psychological well-being	.629	.622	.386	.583

All correlations significant at p < .001.

### Relationship with EQ-5D

The validity of the PIPP Impact subscales was assessed by investigating the relationship with appropriate corresponding EQ-5D items administered to participants. The PIPP Self-care subscale was compared with the EQ-5D self care item. Due to the small numbers of respondents in the 'unable' response category of the EQ-5D Self-care item, respondents were collapsed into two categories: (1) no problems (N = 129), and (2) some problems or unable to care for self (N = 81). Mann-Whitney tests revealed significant differences between the two groups on the PIPP Impact Self-care subscale (z = -8.28, p < .001). The mean rank scores on the PIPP Impact Self-care subscale was higher for the respondents classified as having self-care problems on the EQ-5D (149 vs 78), supporting the validity of the PIPP Impact Self-care subscale.

Kruskal-Wallis tests were conducted to compare the PIPP Impact Mobility subscale scores with responses on the EQ-5D Mobility item (no problem, some problems, confined to bed), although the majority of participants indicated the middle category on the EQ-5D (i.e. 83%). There was a statistically significant difference (chi-square = 22.53, df = 2, p < .001). Mean ranks for each group were in the expected direction with those 'confined to bed' showing the highest PIPP Impact Mobility scores (154 vs 105 vs 56).

Kruskal-Wallis tests were conducted to compare PIPP Impact Participation scores for respondents in each of the three response categories to the EQ-5D item 'Usual Activities' (no problems, some problems, unable to perform). There was a statistically significant difference (chi-square = 33.87, df = 2, p < .001), with mean ranks for each group in the expected direction (i.e. those 'unable to perform' showing the highest PIPP Participation impact scores: 138 vs 109 vs 68).

To assess the construct validity of the PIPP Impact Psychological Well-being subscale, scores were compared to those obtained for the EQ-5D Anxiety/Depression item. Kruskal-Wallis tests revealed a statistically significant difference in scores (chi-square = 22.62, df = 2, p = .001). Mean ranks for each group were in the expected direction with those indicating extreme anxiety and/or depression on the EQ-5D also showing the highest PIPP Impact Psychological Well-being mean rank scores (127 vs 107 vs 73).

## Discussion

The aim of this study was to validate the use of the PIPP among people with a disability in Thailand. The PIPP was initially developed as a multidimensional generic measure of the impact and distress of health conditions from the individual's perspective, and has been validated in an Australian sample [9]. The initial validation of PIPP in Australia revealed adequate psychometric properties for five subscales (Self-care, Mobility, Participation, Relationships, Psychological Well-being) for both impact and distress. One of the difficulties in translating Western-developed concepts from English into different languages is ensuring congruent meanings, particularly in the case of abstract nouns. In Thai, the term 'distress' translates to '*took*' or 'suffer', while impact is '*pon-kratop*' or effect [33,34]. These two words have a similar meaning in Thai, although distress connotes cause; impact is consequence. Initial analysis suggested that participants in the current study did not necessarily differentiate between the terms 'impact' and 'distress.' Preliminary analysis indicated concordant scores for impact and distress on an item-by-item basis, suggesting a lack of differentiation of the concepts. An alternative explanation is the trend towards consistency in responses, i.e. reporting would reflect the expectation that any illness that had specified impact would have a similar level of distress. This latter interpretation is consistent with the tendency for Thai to select the midpoint on Likert scales, reflecting cultural values of harmony and equanimity ("not good, not bad").

The similarity of Impact and Distress responses from Thai participants contrasts with Australians, who were able to distinguish the direct impact (function) of their health condition and the distress (emotional response to loss of function) caused by it. For this paper, we decided that it would only be appropriate to attempt to validate the PIPP Impact subscales at this stage for use in the Thai sample, and not the Distress subscales. Further research is required to explore the understanding of differences between impact of health problems and the distress caused by this impact, in the Thai context.

For all PIPP Impact subscales it was necessary to collapse the original 6-point response scale to a 4-point response scale. For most of the items disordered thresholds were detected which suggested that, while participants could consistently differentiate the two extreme response points, they were not able to reliably distinguish among the four response points in the centre of the scale. Although a number of alternatives were considered, the most suitable action was to recode all items from 012345 to 011223. It may be appropriate in future administrations of the scale for the four response points to be labeled (e.g. *no impact, mild impact, moderate impact, extreme impact*) to assist respondents to distinguish more clearly between response options This change in scale format, however, would require further psychometric testing.

The rescoring strategy used in this study was different to the rescoring adopted in the previous Australian study, which was collapsed to a simpler 3-point response scale [9]. The reduction of the 6-point to the 3-point scale used in the Australian validation resulted in adequate, but not ideal, person separation reliability values (less than .80). For this Thai sample a 4-point response scale appears to be most appropriate, resolving disordered thresholds, while retaining good person separation values (above .80). It is recommended that at this stage of the development of PIPP, no universal change to the response scale be made. Rather, further research investigating the response format across different health conditions and different cultural contexts is required. Future studies involving the pooling of data from multiple sites with the anchoring of scores on a common metric, would allow further exploration of the stability of the PIPP response format and item content across different samples.

In the current Thai study the five PIPP Impact subscales showed adequate psychometric properties, with all demonstrating fit to the Rasch model. All subscales showed adequate person separation reliability. Only one misfitting item was identified (item 10 from Mobility: '*carry*') requiring removal from the subscale. No DIF was found for either gender or age, except for *participation in family activities *in the Participation subscale. Specifically, men indicated a greater likelihood of endorsing this item than women. Given that removal of the item would have resulted in an undesirable reduction in the person separation reliability of the scale, and that the DIF was relatively minor, the item was retained in the subscale. One possible explanation for the DIF for this item is the interpretations of the term 'family activity'. In traditional Thai settings, earning an income and providing for the family is considered an important responsibility of men and therefore, likely to be one of the major ways in which men evaluate their 'participation in family activities' [35]. In the qualitative part of our study, many of the men indicated that they were not able to work in the fields or participate in wage labor because of their impairment, subsequently impacting on their endorsement of the item.

The construct validity of the PIPP was assessed using the Thai version of the EQ5D, which measures how much difficulty a person has in relation to various domains. Those who indicated greater difficulty in regard to Self-care, Mobility, and Participation as measured by EQ5D were also more likely to report higher impact in the equivalent PIPP subscale. Additionally, those who indicated higher levels of anxiety/depression on the EQ5D also reported higher levels of impact on the PIPP Psychological well-being subscale.

## Conclusion

The purpose in developing the PIPP was to construct a suitable tool for measuring the impact and distress of a health condition from the individual's perspective, using the current ICF model as a framework. Consistent with the biopsychosocial model underlying the ICF, the PIPP provides a tool that examines the physical, social, and psychological impact of health. While both the Impact and Distress scales have been validated for use in the Australian context, social, linguistic and cultural factors influence the use of instrumentation in other settings. The results of this study support the psychometric properties of the PIPP Impact subscales in adults with locomotor disability in Thailand. Further work is needed to assess the difference between the Impact and Distress subscales of the PIPP and to test the generalizability of these findings in larger studies, involving different health conditions and cultural settings. The optimal number of response points for the scale also requires further investigation.

## Abbreviations

**DIF: **Differential Item Functioning; **ICF: **The International Classification of Functioning, Disability and Health; **PIPP: **Perceived Impact of Problem Profile; **PSI: **Person Separation Index; **RESILIENCE: **Research into Social Inclusion, Locomotor Impairment and Empowerment through Networking, Collaboration and Education; **VAS: **Visual Analogue Scale; **WHO: **World Health Organization.

## Competing interests

The author(s) declare that they have no competing interests.

## Authors' contributions

The literature review was undertaken by RM, the statistical analyses were conducted by both RM and JP, the study was designed by LM, SC, and JP, and the data collection was managed by LM and SC. All authors contributed to the preparation of the article and approved the final manuscript.
